# Host Organelle Interactions Facilitate Cholesterol Acquisition by *Trypanosoma cruzi* Amastigotes

**DOI:** 10.1111/jeu.70027

**Published:** 2025-07-20

**Authors:** Carolina de Lima Alcantara, Miria Gomes Pereira, Wanderley de Souza, Narcisa Leal da Cunha‐e‐Silva

**Affiliations:** ^1^ Centro de Pesquisas em Medicina de Precisão, Instituto de Biofísica Carlos Chagas Filho Universidade Federal do Rio de Janeiro Rio de Janeiro Brazil; ^2^ Centro Nacional de Biologia Estrutural e Bioimagem Universidade Federal do Rio de Janeiro Rio de Janeiro Brazil

## Abstract

Chagas disease, caused by the protozoan *Trypanosoma cruzi*, is a major neglected disease in Latin America. The amastigote, the replicative intracellular form, is essential for infection persistence in vertebrate hosts. These forms exhibit remarkable adaptability, modulating metabolism and growth according to host cell resource availability. Lipid metabolism plays a critical role in amastigote development, with a strong dependence on host‐derived lipids, particularly cholesterol. Although *T. cruzi* can synthesize some sterols and fatty acids, it also scavenges essential lipids from the host. Changes in host cholesterol metabolism, possibly via SREBPs, may increase intracellular cholesterol levels and promote parasite growth. However, the mechanisms of cholesterol acquisition by amastigotes remain unclear. Here, we investigated cholesterol trafficking from host cells to amastigotes using a fluorescent cholesterol analog. Through confocal and volume electron microscopy, we demonstrated cholesterol uptake by amastigotes, characterized uptake kinetics, and confirmed its importance for parasite development. We also revealed close contact between the host endoplasmic reticulum and the amastigote plasma membrane, consistent with membrane contact sites. Furthermore, we showed that amastigotes can internalize ER‐ and Golgi‐derived host markers, suggesting a potential route for acquisition of host molecules. These findings provide new insights into lipid acquisition strategies by intracellular *T. cruzi* amastigotes.

## 1 Introduction

Chagas disease is one of the most prevalent neglected diseases in Latin America (Ribeiro 2024) and is caused by infection with the protozoan parasite *Trypanosoma cruzi*. *T. cruzi* 's life cycle takes place in vertebrate and invertebrate hosts and comprises proliferative and infective forms that are highly adaptable to their hosts to maintain the infection. In the vertebrate host, amastigote forms are responsible for parasite replication and persistence of the infection in chronically infected patients.

Amastigotes live free in the host cell cytosol, having access to a myriad of macromolecules from the host and host cell organelles. Scavenging nutrients from hosts is central to a parasitic lifestyle. One of the parasites' major mechanisms of nutrient salvage is the presence of plasma membrane transporter proteins (Landfear 2011). Some works have suggested the participation of transporters at the cell surface of amastigotes that may help in micronutrient and ion acquisition to sustain their growth and replication (Pagura et al. 2020; Campagnaro et al. 2018; Dumoulin and Burleigh 2021). However, amastigotes also possess an active machinery for endocytosis: a specialized membrane invagination called the cytostome‐cytopharynx complex, through which macromolecules are endocytosed (Alcantara et al. 2021, 2014). It has been shown that amastigotes recently released from the host cell cytosol by mechanical disruption can endocytose albumin and transferrin added to the extracellular incubation medium (Alcantara et al. 2021; Batista et al. 2015). Labeling cytosolic host proteins with a fluorescent probe showed that amastigotes cannot do bulk endocytosis of cytosolic host proteins, suggesting that other mechanisms control the endocytosis of host cell components (Alcantara et al. 2021).

Although many groups are involved in understanding how the parasite manipulates cell traffic for its benefit, the lipid traffic between the host and amastigotes is only slightly comprehended. Besides, some amastigotes are found in dormancy during the chronic stage (Ward et al. 2020; Sanchez‐Valdez et al. 2018); therefore, additional pharmacological strategies are needed to control parasite proliferation.

Biochemical studies have shown that *T. cruzi* epimastigotes, the replicative forms found in the insect mid‐gut, can synthesize de novo their own sterols, mainly ergostane‐type sterols, as well as scavenging cholesterol from the host (Urbina et al. 1995) via endocytosis of lipoproteins. Epimastigotes grown in axenic culture can endocytose LDL and accumulate the released cholesterol in lipid inclusions in the reservosomes or lipid droplets in the cytosol, being able to mobilize these stocks when necessary (Pereira et al. 2011, 2018, 2015).

Amastigotes are capable of de novo synthesis of sterols, including ergosta‐7‐en‐3β‐ol, ergosta‐7,24(241)‐dien‐3β‐ol (episterol), and 24‐ethyl‐7‐en‐cholesta‐3β‐ol, as shown by Liendo et al. (1999), as well as fatty acids (Li et al. 2016). However, although these endogenous pathways contribute to the parasite's sterol pool, they are not sufficient to compensate for the absence of host‐derived cholesterol. This is evidenced by the significant presence of mammalian sterols such as cholesterol and cholesta‐5,24‐dien‐3β‐ol (desmosterol) in amastigotes, suggesting active lipid scavenging from the host. Notably, cholesterol alone accounts for approximately 80% of total sterols by weight in amastigotes (Liendo et al. 1999), highlighting the central role of host cholesterol for parasite viability and intracellular development.

Omics works in the last decade have provided important clues about host cell and parasite cellular pathways modulated during infection (Gazos‐Lopes et al. 2017; Caradonna et al. 2013; Li et al. 2016). In this context, it has been shown that amastigote growth and replication are highly dependent on the lipid metabolism of the host cell (Gazos‐Lopes et al. 2017; Caradonna et al. 2013). *T. cruzi* infection can lead to changes in the cholesterol metabolism of the host by increasing intracellular cholesterol levels (Johndrow et al. 2014), probably by upregulation of the SREBPs (sterol regulatory element binding proteins) that regulate lipid homeostasis (Li et al. 2016), favoring parasite development. However, direct evidence for the role of cholesterol in parasite growth and development is absent.

Despite the biochemical evidence pointing to a role of host lipids in the metabolism of intracellular amastigotes, the mechanisms by which amastigotes scavenge these lipids are unclear. In this work, using a fluorescent cholesterol analog, we could follow cholesterol traffic from the host cells to the amastigotes using confocal fluorescence microscopy. We were able to define the kinetics of cholesterol acquisition by amastigotes and the possible host organelles involved in the traffic. Moreover, we demonstrated that cholesterol is important for amastigote growth and development. Ultrastructural analysis by electron tomography and FIB‐SEM (*Focused Ion Beam‐Scanning Electron Microscopy*) showed the presence of contact sites between the parasite and host ER and Golgi complex that might be responsible for the acquisition of cholesterol by amastigotes and provided important information about these organelles remodeling in the course of *T. cruzi* infection.

## 2 Materials and Methods

### 2.1 Cell Culture and Parasites

Human Foreskin Fibroblasts (HFF‐1, SCRC‐1041, ATCC) were maintained as subconfluent monolayers in Dulbecco's modified Eagle's medium (DMEM, Gibco, Grand Island, NY) supplemented with 10% fetal bovine serum (FBS, Gibco, Grand Island, NY), 2 mM L‐alanyl‐L‐glutamine dipeptide in 0.85% NaCl (Glutamax, Gibco, Grand Island, NY) and 1% penicillin–streptomycin (Gibco), at 37°C under 5% CO_2_. Tissue culture *Trypanosoma cruzi* trypomastigotes from clone Dm28c were used to infect HFF1 cells with a MOI of 10:1. The development of intracellular amastigotes was followed over time.

### 2.2 Serum Delipidation

FBS was delipidated according to Cham and Knowles (1976). The delipidated fetal bovine serum (dFBS) was sterilized by filtration with a 0.22 μm membrane (Millex‐GV, Millipore S.A., Molsheim, France).

### 2.3 TopFluor Cholesterol Preparation and Complexation With LDL Particles

TopFluor Cholesterol (Avanti Polar Lipids, catalog number 810255) was dissolved in pure ethanol at a concentration of 1 mg/mL. Low‐density lipoprotein (LDL) was purified from fresh human plasma as described by Chapman et al. (1981) (Chapman et al. 1981) with some modifications. 100 μg of TopFChol was mixed with 12 mL of human cell‐free plasma followed by KBr addition to adjust the density to 1.3 g/mL. The plasma was added to a centrifuge tube with 20 mL of saline solution (150 mM NaCl + 1 mM EDTA). This material was ultracentrifuged at 150.000 *g* in a vertical angle Beckman VTi 50 rotor (Beckman Coulter Inc., Fullerton, CA, USA) at 4°C for 12 h. The LDL fraction was localized and removed. KBr was added again to the LDL fractions to adjust the density to 1.2 g/cm^3^, and the material was ultracentrifuged at 150.000 *g* in the same rotor at 4°C for more than 12 h. The purified lipoproteins complexed to TopFChol were extensively dialyzed against PBS with 1 mM EDTA.

### 2.4 Generation of Amastigotes Expressing mSC::MyoF Cell Line

*T. cruzi* cell line expressing mSC::MyoF was generated from Cas9‐expressing epimastigotes, produced as described in Alves et al. (2022) but using a pPOTc blast mSC::myc plasmid. mSC::MyoF epimastigotes were submitted to in vitro metacyclogenesis as described before (Contreras et al. 1985) to generate mSC::MyoF metacyclic trypomastigotes. These forms were used to infect HFF cells to produce mSC::MyoF intracellular amastigotes.

### 2.5 TopFChol Assays in Infected Cells

For experiments where TopFChol was added directly to the culture medium, cells were infected with trypomastigotes (WT or mSC::MyoF) (MOI 10:1) in a medium containing 10% FBS for 4 h. Cells were washed to remove parasites that did not enter, and incubation with TopFChol was made 24 h later. TopFChol, at a final concentration of 10 μM, was diluted in a culture medium containing 10% dFBS before incubation with cells. For incubations with TopFChol loaded into LDL particles, infected cells were prepared as described above. LDL‐TopFChol, at a concentration of 100 μg/mL, was added to the culture medium containing 10% dFBS. At the end of the incubation period, cells were washed with PBS and fixed with 4% (v/v) methanol‐free formaldehyde in PBS.

### 2.6 Quantification of TopFChol Internalization by Amastigotes

Incubation of infected cells with LDL‐TopFChol or free TopFChol was performed as described above. Samples were imaged in a Zeiss Elyra PS.1 confocal laser microscope equipped with an ACS APO 63.0x1.40 OIL DIC objective. Laser lines 405, 488, 543, and 633 were used. *Z*‐stack series were acquired with the Zen software (Zeiss) with a frame averaging of 2 and a step size of 0.3 μm. For quantification of amastigote internalization of TopFChol, amastigotes were divided into two halves (Figure 3a), and labeling was classified based on the presence of the tracer at the amastigote's cell anterior, anterior and posterior, or no labeling. Two hundred amastigotes were counted for each condition (from two independent experiments). Statistical analysis was performed by two‐way analysis of variance (ANOVA) followed by Bonferroni's multiple comparison test, using the Graph Pad Prism 9.5.1 software (La Jolla, CA, USA). All quantification data represent mean and standard deviation values. Results were considered statistically significant when *p* < 0.05.

### 2.7 Amastigote Proliferation

HFF cells were infected with trypomastigote (MOI 10:1), incubated for 4 h, washed to remove parasites that did not enter cells, and incubated for more than 20 h in medium supplemented with 10% FBS. Afterward, the medium was changed, and cells were incubated in medium containing 10% FBS (control), 10% dFBS, or 10% dFBS + 10 μM of TopFChol. Cells were fixed 24 h later (48 hpi) with 4% (v/v) formaldehyde in PBS, pH 7.2, and stained with DAPI. Three hundred host cells were counted for each condition (two independent experiments in triplicate) using the Cell Counter plugin of ImageJ. Parasite/cell and percentage of infection (% infection) were normalized by the control sample. Graph plotting and result analysis were performed using the one‐way ANOVA with Post Hoc Holm‐Šídák's multiple comparisons test, as adequate. The analyses were carried out using the GraphPad Prism 9.5.1 software (La Jolla, CA, USA).

### 2.8 Fluorescence Microscopy

Infected or non‐infected HFF1 cultures, incubated or not with TopFChol, were fixed with 4% (v/v) formaldehyde in PBS, pH 7.2. For immunolabeling experiments, cells were washed in PBS, pH 7.2 to remove the fixative, incubated with 150 mM ammonium chloride for 15 min, and incubated with a blocking solution (3% BSA, 0.1% saponin in PBS pH 8) for 1 h. Primary antibodies were diluted in the blocking solution and incubated for 1 h, followed by incubation with secondary antibodies, also diluted in the blocking solution, and incubated for 1 h. Samples were DAPI (4′, 6‐diamidino‐2‐phenylindole; Thermo Fisher) stained and mounted on microscopy slides using Prolong Diamond antifade Mountant (Thermo Fisher). Primary antibodies used were: anti‐PDI (Thermo Fisher—PA582640); anti‐TGN‐38 (Sigma Aldrich—T9826); anti‐Lamp1 (Thermo Fisher—PA1654A); and anti‐Ssp4 (monoclonal 2C2; Andrews et al. 1987), kindly provided by Dr. Renato Mortara (Unifesp, São Paulo, Brazil). Secondary antibodies used were Alexa Fluor 594 Goat Anti‐Rabbit or Anti‐Mouse IgG (Thermo Fisher), and Alexa Fluor 647 Goat Anti‐Mouse IgG (Thermo Fisher).

Alternatively, cells were incubated with Cell Light ER‐RFP or Cell Light Golgi‐RFP reagents (Thermo Fisher) overnight, according to the manufacturer's instructions, washed in sterile PBS, and then incubated with TopFChol, under the same conditions described above, if that was the case. Cells were fixed as described above. All samples were imaged in a Zeiss Elyra PS.1 confocal laser microscope equipped with an ACS APO 63.0x1.40 OIL DIC objective. Laser lines 405, 488, 543, and 633 were used. *Z*‐stack series were acquired with the Zen software (Zeiss) with a frame averaging of 2 and a step size of 0.3 μm.

### 2.9 Isolation of Intracellular Amastigotes and Cell Cytometry Assay

HFF cells were infected with trypomastigote (MOI 10:1), incubated for 4 h, washed to remove parasites that did not enter cells, and incubated for more 44 h in medium supplemented with 10% FBS and the Cell Light ER‐RFP, according to the previous section. Amastigotes were isolated from host cells as described previously (Alcantara et al. 2021) and the amastigote‐enriched fraction was fixed and labeled with anti‐SSP4 antibody as already described. The percentage of amastigotes containing ER‐RFP labeling was measured by cell cytometry, by first defining the gate where the amastigotes were localized (SSC × FSC) and then counting 40,000 events. Reads were done in a BD Accuri C6 equipment.

### 2.10 Sample Preparation for Transmission Electron Microscopy (TEM)

Infected or non‐infected cultures were fixed by using 2.5% (v/v) glutaraldehyde in 0.1 M cacodylate buffer, pH 7.2, for 1 h at room temperature, post‐fixed using an osmium‐thiocarbohydrazide‐osmium (OTO) protocol (Willingham and Rutherford 1984; Alcantara et al. 2014), dehydrated in an acetone series, and embedded in epoxy resin. Samples were cut in ultrathin sections in a Leica EM UC7 Ultramicrotome and stained post‐embedding with 5% (w/v) uranyl acetate and lead citrate. Samples were imaged in a Tecnai Spirit electron microscope (Thermo Fisher) operating at 120 kV or in a Hitachi HT 7800 (Hitachi) operating at 120 kV.

### 2.11 Immunogold Labeling

Infected or non‐infected cultures incubated with TopFChol for 24 h as described above were fixed with 4% (v/v) methanol‐free formaldehyde, 0.2% (v/v) glutaraldehyde in 0.1 M cacodylate buffer, pH 7.2, for 1 h at room temperature. Samples were dehydrated in an ethanol series, infiltrated, and embedded in LR White acrylic resin. Samples were cut into ultrathin sections in a Leica EM UC7 Ultramicrotome, and sections were collected in nickel grids. Grids were blocked with a blocking buffer (1.5% BSA in PBS pH 8) for 1 h, then incubated with BODIPY FL Polyclonal Antibody (Thermo Fisher) diluted 1:100 in the blocking buffer for 1 h, and with secondary antibody goat anti‐rabbit IgG Nanogold (Thermo Fisher) diluted 1:100 in blocking buffer for 1 h. After several washes in water, nanogold labeling was enhanced using the Pierce Silver Stain Kit (Thermo Fisher), according to the manufacturer's instructions. Grids were not post‐stained and were observed in a Hitachi HT 7800 operating at 120 kV.

### 2.12 Electron Tomography

Samples processed for TEM were cut in 200‐nm‐thick serial sections using a Leica EM UC7 Ultramicrotome. Sections were collected onto formvar‐coated copper slot grids and post‐embedded stained as described before. Single or dual‐axis tilt series (±65° with 1° increment) were produced in a Tecnai Spirit electron microscope (Thermo Fisher) operating at 120 kV and coupled to a 2 k × 2 k pixel CCD camera. Tilt series were reconstructed by weight back projection using ETOMO software from the IMOD package (Kremer et al. 1996).

### 2.13 Focused Ion Beam‐Scanning Electron Microscopy

Samples prepared for TEM were trimmed and glued to an SEM stub using carbon tape and metalized with gold. Samples were imaged using an Auriga dual‐beam microscope (Zeiss) equipped with a gallium‐ion source for focused‐ion‐beam milling, a field‐emission gun, and an in‐lens secondary electron detector for SEM imaging. The cross‐sectional cut was made at ion beam currents of 2.0 uA and an accelerating voltage of 30 kV. Backscattered electron images were recorded at an accelerating voltage of 1.8 kV and a beam current of 0.8 nA in the immersion lens mode using a CBS (Concentric Back Scatter) detector. A series of backscattered electron images were recorded in “slice‐and‐view” mode at a magnification of 15 K with a pixel size of 3.5 nm and a milling step size of 30 nm. After image capture, backscattered electron images had their contrast inverted to resemble conventional TEM images. FIB‐SEM serial images were aligned using ETOMO software from the IMOD package.

### 2.14 Tridimensional Reconstruction

Serial tomograms and serial sections obtained in FIB‐SEM were segmented and rendered using 3DMOD from the IMOD package.

## 3 Results

### 3.1 Amastigotes Internalize Extracellular‐Derived Cholesterol

**FIGURE 1:**
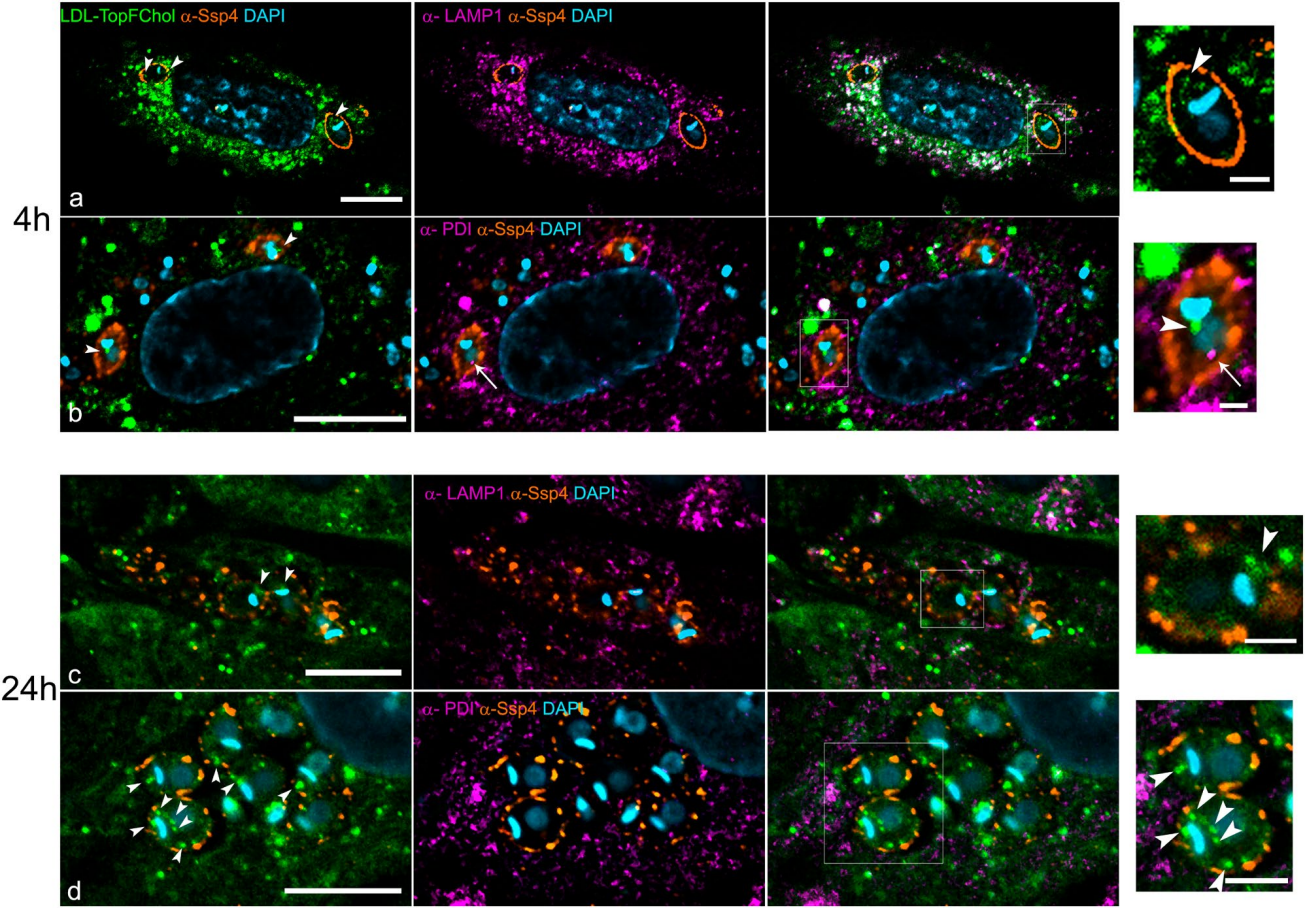
Dynamics of TopFChol internalization by intracellular amastigotes through incubation with LDL‐TopFChol. HFF1 cells were infected with trypomastigotes and, 24 h after, were incubated with LDL‐TopFChol in a growth medium supplemented with 10% of dFBS. Single‐plane fluorescence images from the *z*‐stack confocal series are shown. (a, b) Cells were incubated with LDL‐TopFChol (green) for 4 h, fixed and co‐stained with anti‐Lamp1 (to label lysosomes) or anti‐PDI (to label ER) antibodies (magenta). TopFChol signal colocalized with anti‐Lamp1 staining (a) but not with anti‐PDI (b). Intracellular TopFChol labeling was observed inside amastigotes in punctual locations at the cell anterior (a) and posterior to the kinetoplast (b). Anti‐Ssp4 antibody was used to label the amastigote cell membrane (orange) and DAPI (blue) stained cells DNAs. On the note, an anti‐PDI signal was observed inside amastigotes at 4 h at the post‐nuclear location (b, arrow). (c, d) LDL‐TopFChol was incubated with infected cells for 24 h. TopFChol no longer colocalizes with anti‐Lamp1 labeling (c), instead, showed a spread labeling through the cytoplasm, partially colocalizing with the anti‐PDI signal at the ER (d). At this time point, more amastigotes showed intense punctual intracellular labeling for TopFChol (arrowheads). Insets displayed next to each panel show magnified views of the regions outlined by white rectangles. Bars: 10 μm; insets 2 μm.

To follow the traffic of cholesterol from the host cell to the amastigotes, we first used LDL particles loaded with a fluorescent cholesterol analog, TopFluor Cholesterol (TopFChol) diluted in delipidated FBS (dFBS), to favor cholesterol acquisition by the cells. Infected host cells were incubated with LDL‐TopFChol for different times: a short incubation of 4 h and a longer incubation of 24 h. After 4 h of incubation, the LDL‐TopFChol signal was found adjacent to or partially overlapping with LAMP1‐positive compartments, indicating a possible interaction between the cholesterol‐rich vesicles and endolysosomal structures (Figure 1a). At this time point, no major colocalization of the TopFChol signal with the host ER was observed (Figure 1b). At this short incubation time, it was already possible to see punctual labeling of the TopFChol inside amastigotes, especially at the anterior region of the parasites (Figure 1a,b). After 24 h of incubation, the TopFChol signal derived from endocytosed LDL no longer showed prominent overlap with lysosomal markers (Figure 1c), and its distribution appeared more diffuse, resembling patterns typically associated with incorporation into internal host membranes (Figure 1d). This observation is consistent with previously described LDL‐derived cholesterol trafficking in mammalian cells (reviewed in Ikonen and Olkkonen (2023)). At this point, more amastigotes showed spotted TopFChol signal inside their cytosol, mostly at the parasite's anterior region, close to the main endocytic portals, the flagellar pocket (FP), and the cytostome‐cytopharynx complex (Figure 1c,d). Of note, we observed labeling for the luminal ER protein PDI (*Protein Disulfide Isomerase*) inside amastigotes (Figure 1b).

**FIGURE 2:**
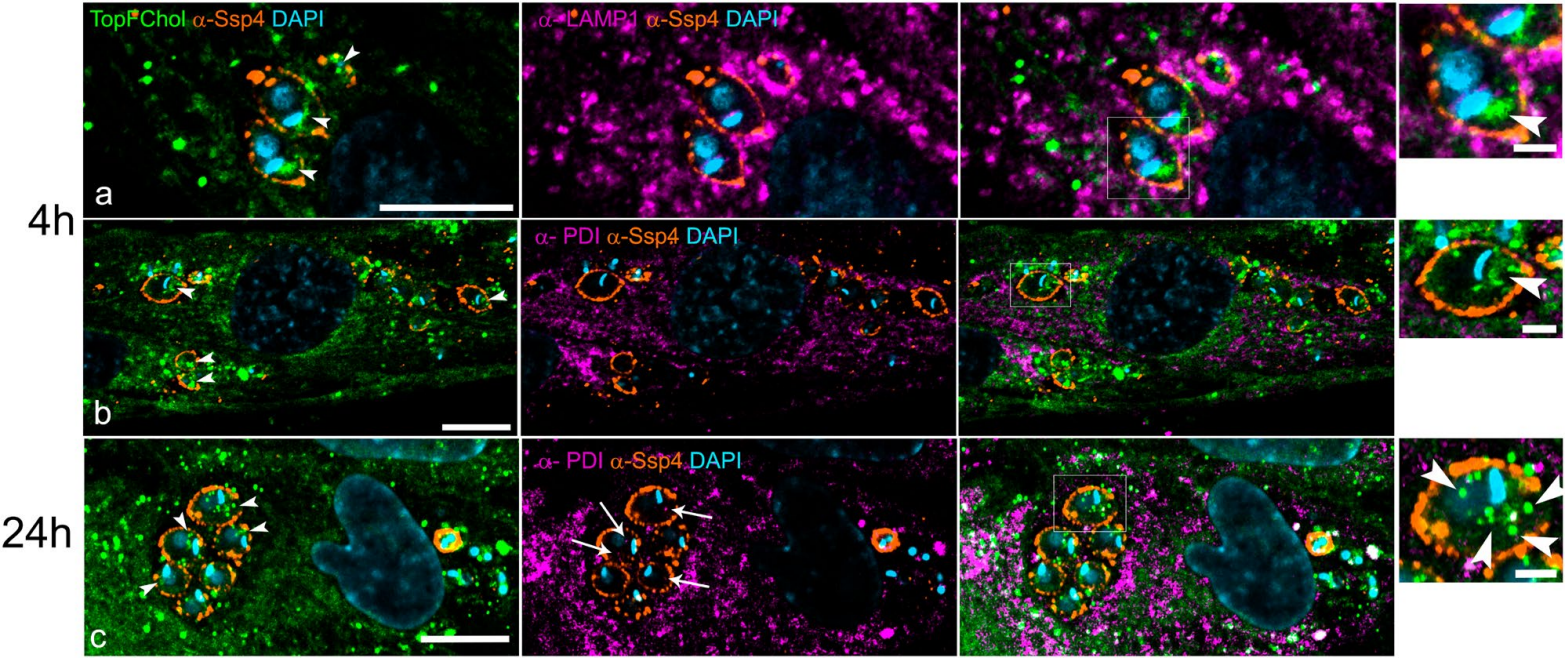
Dynamics of TopFChol internalization by intracellular amastigotes through incubation with TopFChol directly into the medium. HFF1 cells were infected with trypomastigotes and, 24 h after, TopFChol was added directly to the medium supplemented with 10% of dFBS. Single‐plane fluorescence images from the *z*‐stack confocal series are shown. (a, b) Cells were incubated with TopFChol (green) for 4 h, fixed and co‐stained with anti‐Lamp1 (a) or anti‐PDI (b) antibodies (magenta). An intense TopFChol signal can be observed inside the amastigotes (arrowheads in a, b). No colocalization of TopFChol signal and Lamp1‐positive compartments was observed (a) but instead seemed to colocalize with PDI‐positive compartments (b). (c) TopFChol was incubated with infected cells for 24 h. Amastigotes showed intense punctual TopFChol labeling along the anterior–posterior region (arrowheads). Labeling for PDI was observed inside amastigotes in regions that colocalized with the TopFChol signal (arrow). Anti‐Ssp4 antibody was used to label the amastigote cell membrane (orange) and DAPI (blue) stained cells DNAs. Insets displayed next to each panel show magnified views of the regions outlined by white rectangles. Bars: 10 μm; insets 2 μm.

We investigated other routes of cholesterol traffic into the host cell by incubating the TopFChol directly in the culture medium. In this condition, most cholesterol molecules should be inserted into the plasma membrane (PM) where they can be internalized through endocytosis or sequestered into the ER via PM‐ER contact sites, restoring the cholesterol levels at the PM (Ikonen and Olkkonen 2023). We observed that, after 4 h of incubation, several amastigotes already showed spots of TopFChol inside the parasites, preferably at the anterior region (Figure 2a,b). After 24 h of incubation, a stronger TopFChol signal was consistently observed inside the amastigotes (Figure 2c). In some regions, the TopFChol fluorescence appeared near PDI labeling, suggesting a spatial association between internalized cholesterol and host ER‐derived structures.

**FIGURE 3:**
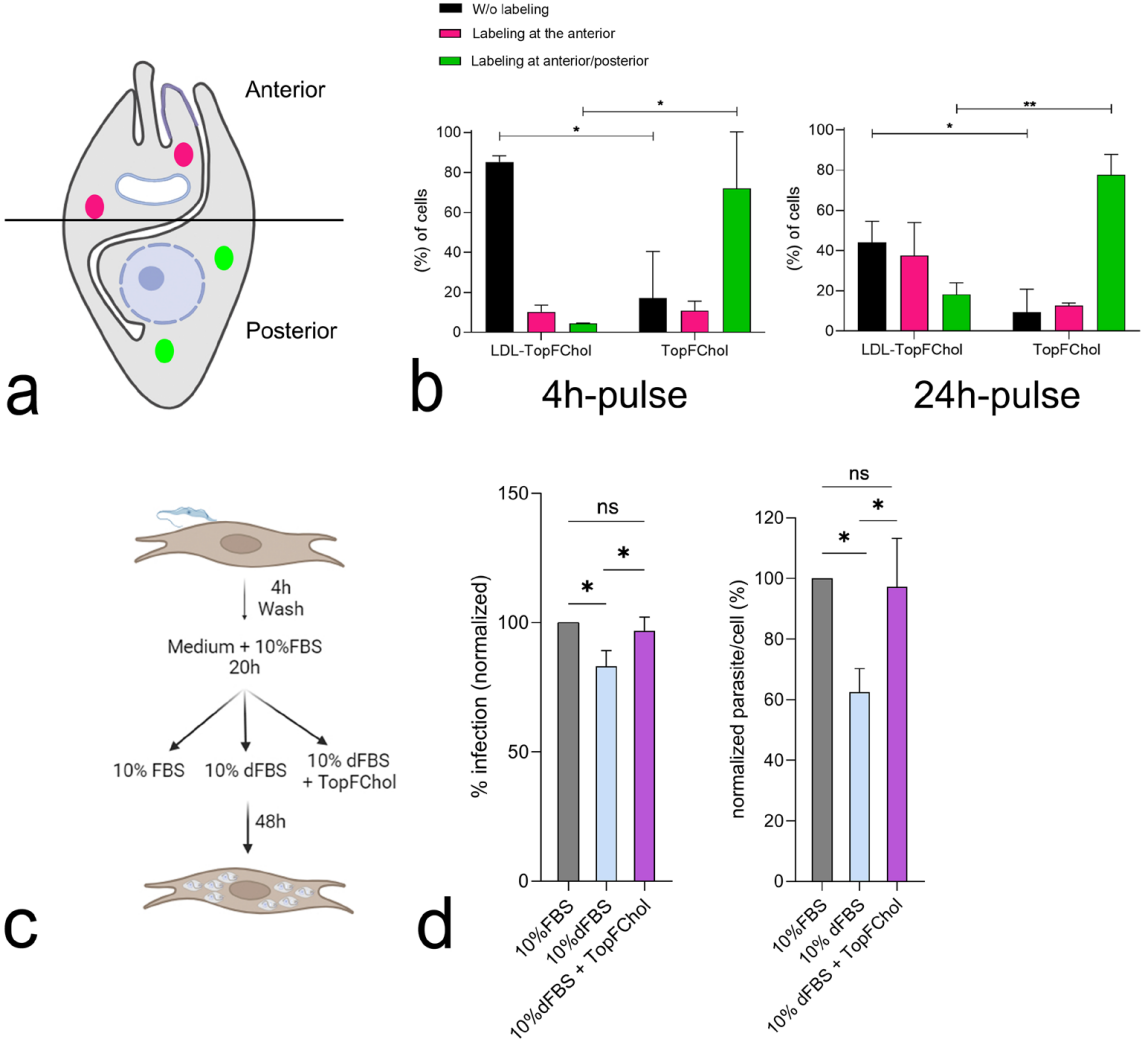
Kinetics of TopFChol internalization by amastigotes and cholesterol effect in amastigote proliferation. (a) Schematic representation of the amastigote division parameter used for the analysis shown in (b). The amastigote cell body was divided into two halves. All labeling localized from the anterior region until the posterior of the kinetoplast was considered to be at the cell's anterior halves (pink bars). All labeling localized from the posterior of the kinetoplast to the posterior tip of the amastigotes was tagged as posterior labeling. Green bars represent labeling at both anterior and posterior regions. (b) HFF1‐infected cells were incubated with LDL‐TopFChol or TopFChol directly in the culture medium for 4 or 24 h. Confocal *z*‐stacks were acquired and TopFChol labeling inside amastigotes was quantified using the criterion shown in (d). 200 amastigotes were counted in each condition. Data represent mean ± SEM values. *p < 0.01 and **p < 0.001 analyzed by two‐way ANOVA followed by Bonferroni. (c) Schematic representation of the experimental design used for determination of amastigotes proliferation in different availability of lipids. (d) Infection efficiency and the numbers of amastigotes per infected cell were determined by counting a total of 300 HFF cells (*n* = 2 in triplicate) in each condition. Data were normalized by the control (10% FBS). Mean ± SD are shown. Data were analyzed using one‐way ANOVA with Post Hoc Holm‐Šídák's multiple comparisons test.

We quantified the presence or absence of TopFChol labeling in amastigotes and the location of the signal inside them (anterior or posterior) at the different times and availability of TopFChol. For that, we divided the amastigotes into two halves, i.e., anterior, from the flagellum tip to the posterior portion of the kinetoplast, and posterior, from the perinuclear region down to the posterior tip (Figure 3a). Two hundred amastigotes were counted for each condition. After 4 h, in cells incubated with TopFChol added directly to the culture medium, a mean of 72% of the amastigotes presented intracellular punctual labeling, both at the cell's anterior and posterior, when compared to only 4% of the amastigotes in host cells incubated with TopFChol loaded into LDL particles (Figure 3b). After 24 h, a mean of 77% of the amastigotes presented labeling of TopFChol both at the cell's anterior and posterior when host cells were incubated with TopFChol in the medium, while a mean of only 18% presented this labeling pattern when host cells were incubated with LDL‐TopFChol. This indicated that Chol was internalized by the amastigotes in both conditions. However, the traffic route of the tracer inserted into the PM, by adding TopFChol directly to the culture medium, was faster than when Chol was provided inside the LDL particles. Next, we evaluated if cholesterol was important for amastigote proliferation. For that, we incubated the infected cells under three different conditions: medium supplemented with 10% whole FBS (control), with 10% dFBS, or with 10% dFBS + TopFChol (Figure 3c). Infected cells and the number of amastigotes/cell were evaluated 48 hpi. We observed a decrease in both infectivity and number of amastigotes/cell when cells were incubated in dFBS, compared with control. However, infectivity and amastigotes/cell were restored to control if TopFChol was added to the medium with dFBS (Figure 3d). This result demonstrated that amastigote proliferation was sensitive to a decrease in cholesterol availability.

**FIGURE 4:**
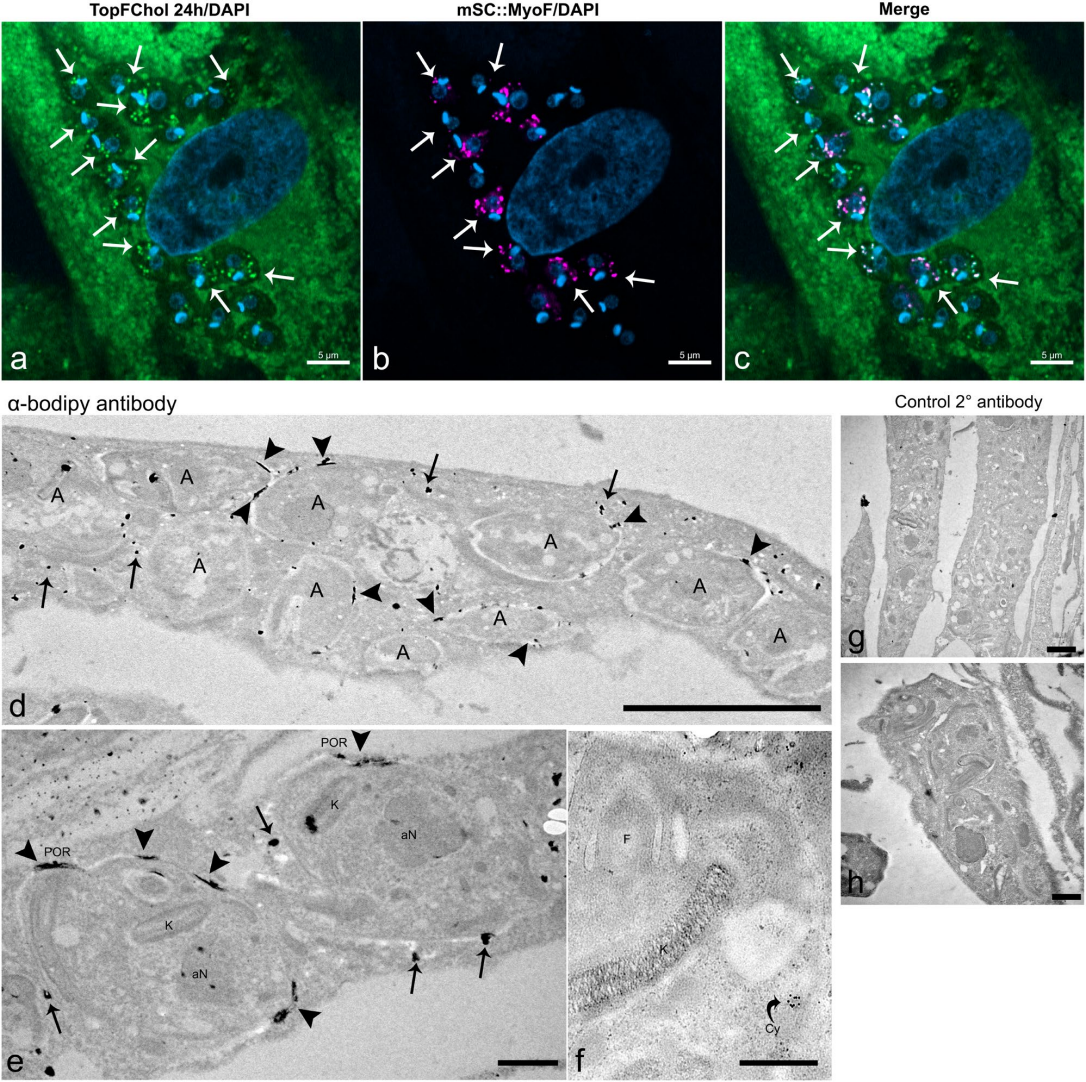
Localization of TopFChol inside amastigotes. (a–c) HFF1 cells were infected with mSC::MyoF trypomastigotes and, 24 h after, TopFChol was added directly to the growth medium supplemented with 10% of dFBS. Single‐plane fluorescence images from the *z*‐stack confocal series are shown. (a) TopFChol signal (green) showed a strong labeling at the amastigote anterior region, close to the kinetoplast (in blue) inside vesicular structures (arrow). (b) Endogenous signal of MyoF tagged with mSC. (c) TopFChol colocalizes with mSC::MyoF positive structures. (d–f) Infected cells, incubated with TopFChol for 24 h were processed for TEM using hydrophilic resin and immunogold labeled with a primary antibody anti‐bodipy moiety of TopFChol and secondary antibody coupled with nanogold. The reaction was silver enhanced. Grids were not post‐stained. (d) Low‐magnification image showing an overview of an infected cell. An intense labeling reaction was observed at puncta around the amastigotes plasma membrane (arrowheads) as well as in regions of the host cytosol (arrows). (e) The strong electron‐dense labeling was observed at the anterior region of the amastigote, specifically at the membrane domain of the preoral ridge (POR) (arrowheads). (f) Some gold particles can be seen at the membrane of the cytopharynx (Cy). (g, h) Images of the same material incubated only with the secondary antibody and silver stained shows the absence of reaction. A, amastigotes; aN, amastigote nucleus; F, flagellum; K, kinetoplast. Bars: a–d—5 μm; e, h—1 μm; f—500 nm; g—2 μm.

The pattern of labeling of the TopFChol inside the amastigotes was peculiar, usually forming a winding or helical shape from the cell's anterior region, where it was more concentrated, to the cell's posterior. This pattern made us ask if TopFChol has been internalized by amastigotes through the cytostome‐cytopharynx membrane domain. For that, we produced parasites expressing the orfan‐myosin MyoF tagged with mScarlet (mSC) fluorescent protein. MyoF was already shown to be part of the cytostome‐cytopharynx complex (Chasen et al. 2020; Alves et al. 2022) and participate in the endocytic process through this structure (Alves et al. 2022). We infected HFF1 cells with amastigotes expressing mSC::MyoF and, after 24 h, we added TopFChol directly to the culture medium and incubated for 24 h. Cells imaged by fluorescence confocal microscopy showed a colocalization between MyoF endogenous signal with TopFChol internalized by amastigotes (Figure 4a,b), in vesicular structures that spread from cell anterior to cell posterior. To further corroborate the participation of the cytostome‐cytopharynx complex in the internalization of TopFChol, we performed immunogold labeling with silver enhancement for the BODIPY moiety of TopFChol in LRWhite sections. We observed an intense labeling at the preoral ridge (POR), flagellar pocket, and in spots along the amastigote plasma membrane, in infected cells incubated with TopFChol for 24 h (Figure 4d,f). Silver enhanced labeling was stronger in areas of the host cell, besides the parasite membrane. We also observed labeling at the membrane of the cytopharynx of an amastigote (Figure 4d).

### 3.2 Role of Host Biosynthetic‐Secretory Organelles in Cholesterol Scavenge by Amastigotes

#### 3.2.1 Amastigote‐ER Contact Sites

The results shown so far point to a probable role of host ER in the traffic of cholesterol for the amastigotes. As mentioned, we observed antibody labeling for host PDI, an ER luminal protein, in punctual locations inside amastigotes. To rule out the possibility of cross‐reactivity of the antibody with amastigote's antigens, we used a transient transfection system, Cell Light ER‐RFP (Thermo Fisher), that uses a baculovirus vector to deliver a fusion construct of ER signal sequence of calreticulin and KDEL (ER retention signal) and the fluorescent tag RFP. Infected cells were incubated overnight with the reagent and the next day incubated with LDL‐TopFChol (Figure S1) or TopFChol directly in the culture medium (Figure S2). In both cases, we observed the same labeling kinetics as before, with 4 h and 24 h of incubation, with a faster delivery of TopFChol to amastigote when TopFChol was added to the culture medium. However, we observed stronger labeling of the Cell Light ER‐RFP inside the amastigotes that colocalized with the TopFChol signal, especially at 24 h (Figure S2), which supports the hypothesis that host‐derived ER components are internalized, although the mechanism of transfer remains to be clarified. We extended the incubation time to 48 h and saw a change in the Cell Light ER‐RFP labeling from reticulated to a more punctate staining pattern, inside and around the amastigotes, whether TopFChol was provided inside LDL or free in the culture medium (Figures S1 and S2).

**FIGURE 5:**
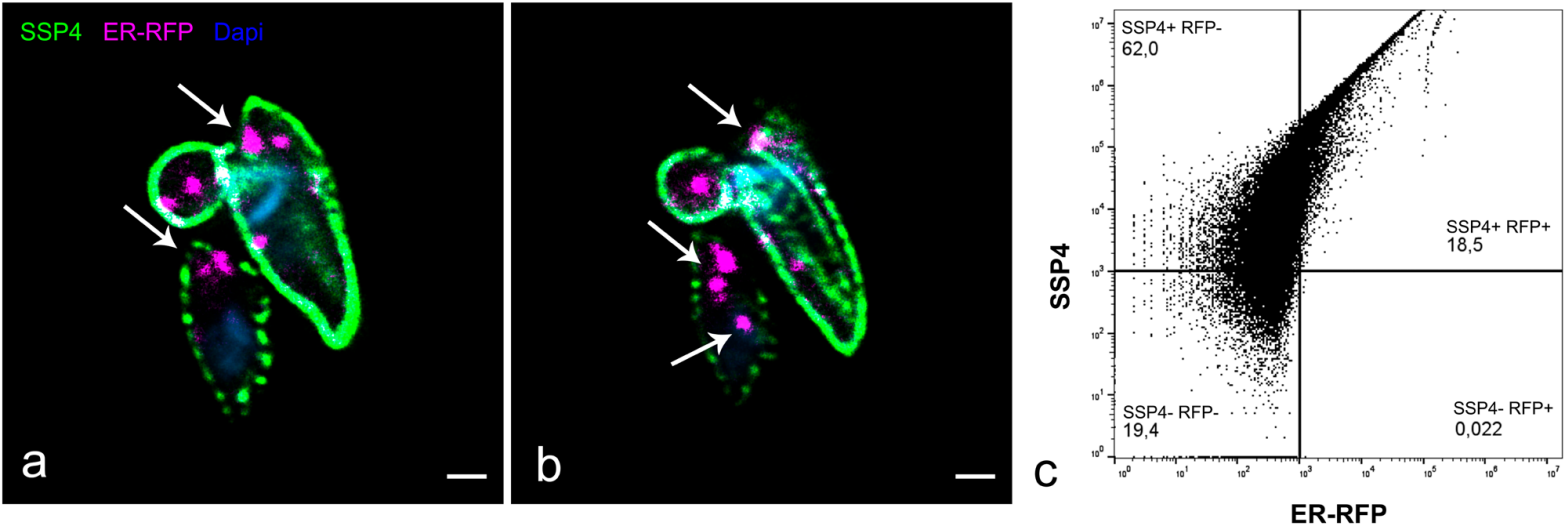
Host ER proteins are internalized by amastigotes. HFF‐1 cells were infected with *T. cruzi* trypomastigotes, washed to remove non‐internalized parasites and incubated with the CellLight ER‐RFP reagent in complete fetal bovine serum (FBS). After 48 h of infection, cultures were lysed, and amastigotes were isolated from the supernatant by differential centrifugation. (a, b) Sequential single planes of a confocal *z*‐stack series of an enriched fraction of isolated amastigotes. The amastigotes were stained with anti‐SSP4 antibodies (green). Images highlight the CellLight ER‐RFP signal (red) in the anterior region of the amastigote (indicated by arrows) (a) and extending along the anterior and posterior regions of the parasite (b). (c) Flow cytometry analysis represented as a dot plot. The analysis shows a population of cells that are positive for SSP4 (amastigotes, green channel) and contain host‐derived ER proteins, as evidenced by the RFP signal (SSP4+ RFP+). 40,000 amastigotes were sorted, and percentages of each population are shown below population label.

As an additional approach to show the internalization of host ER proteins by the amastigotes, we infected host cells with trypomastigotes, added the Cell Light ER‐RFP reagent, and 48 h later we isolated intracellular amastigotes. Enriched fractions with amastigotes were fixed and labeled with anti‐SSP4 antibody. Imaging these samples in the confocal microscope, we could see RFP signal inside isolated amastigotes (Figure 5a,b). Cell cytometry analysis showed that 18% of the isolated amastigotes had ER‐RFP signal (Figure 5c).

To investigate how host–parasite spatial organization may influence cholesterol acquisition by *T. cruzi* amastigotes, we first examined the ultrastructure of infected cells by transmission electron microscopy (TEM). Infected cells at 48 hpi were maintained either in standard medium containing 10% fetal bovine serum (FBS) or in medium supplemented with 10% delipidated FBS (dFBS). In cells incubated with FBS, host ER and lysosomes were frequently observed near the amastigote surface. In several instances, membranes of host organelles and the parasite appeared so closely apposed that their boundaries were indistinct (Figure S3a–c). Additionally, cytosolic material and ER profiles could be visualized at the entrance of the cytostome or near the posterior region of the parasite (Figure S3c–d). In cells grown under lipid‐restricted conditions (dFBS), ER profiles appeared more prominently associated with the amastigotes (Figure S3e). Multiple regions of close apposition were observed, particularly around the anterior region of the parasite (Figure S3f–i). While these 2D images provided initial insights into the spatial organization of host–parasite interactions, they do not resolve the three‐dimensional architecture of membrane contact sites (MCSs).

**FIGURE 6:**
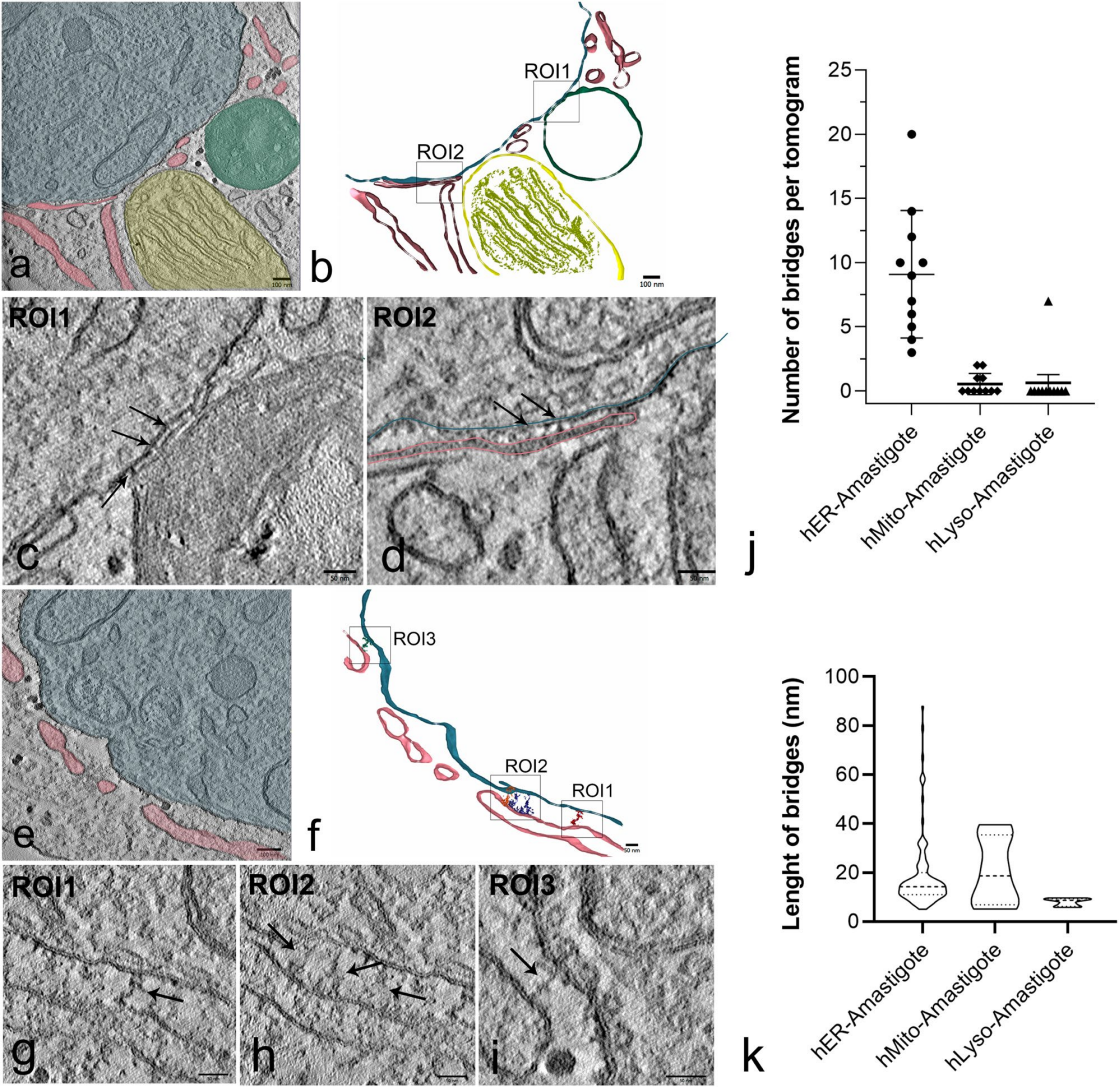
Host organelles exhibit contact sites with amastigote membrane. HFF1 cells were infected with trypomastigotes and incubated with the growth medium supplemented with 10% of complete FBS (control) for 48 h. Cells were then fixed and processed for TEM. (a) A tomogram virtual slice image showing the main host organelles found in the vicinity of amastigotes (colored in blue): Host ER (pink), mitochondria (yellow), and lysosomes (green). Note the proximity of the ER membrane and lysosome membrane to the amastigote. (b) 3D reconstruction of the tomogram showed in (a), highlighting regions of intimate contact between lysosome‐amastigotes (ROI1) and ER‐amastigotes (ROI2). Tomogram images of these ROIs are shown in (c) and (d), respectively. Electron‐dense bridges connecting host lysosome membrane (c) and host ER membrane (d) with the amastigote membrane could be observed (arrows). (e) Virtual slice image of another tomogram showing ER profiles (pink) in the vicinity of the amastigote membrane (blue). In the 3D reconstruction shown in (f), bridges between the two membranes were segmented by threshold and are highlighted in ROIs 1, 2, and 3. These same regions can be observed in the tomogram images shown in (g), (h), and (i), where the bridges are pointed by the arrows. (j) Measure of the number of bridges between each host organelle and amastigote membrane per tomogram (*n* = 11). (k) Measure of the length of the bridges between host organelles and amastigotes. The graph shows the frequency distribution of the bridge's lengths found for each organelle. Bars: a, b, e—100 nm; c, d, f—1–50 nm.

Transmission electron microscopy has long been acknowledged as the benchmark method for visualizing cellular ultrastructure. However, 2D projections lack volumetric context and are limited in their ability to define interorganellar MCSs. To overcome this, we analyzed electron tomograms acquired from 200‐nm‐thick sections of infected cells at 48 hpi, cultured with 10% FBS. A total of 11 tomograms (pixel size: 1.94 nm) were obtained and analyzed. Consistent with our TEM observations, host ER and lysosomes were often found in close apposition to the amastigote plasma membrane (Figure 6a,b). In the 3D reconstructions, discrete bridges could be identified connecting host lysosomes (Figure 6c) and ER (Figure 6d) to the parasite surface. These ER–amastigote contacts displayed a range of morphologies—from short segments where membranes appeared juxtaposed (Figure 6d) to longer tethers suggestive of structured membrane association (Figure 6i). We quantified the number of interorganellar bridges observed between the amastigote membrane and the main host organelles present near the parasite—ER, lysosomes, and mitochondria. As shown in Figure 6j, ER was the most frequent partner, forming an average of ~10 bridges per tomogram. Additionally, we measured the length of these connections (Figure 6k). ER–amastigote bridges displayed a wide range of lengths, from 5 to 80 nm, whereas mitochondria–amastigote bridges were more uniform (5–40 nm), and lysosome–amastigote bridges were consistently short (6–10 nm). Together, these observations highlight the ER as a frequent structural partner of *T. cruzi* amastigotes and reinforce the potential role of interorganellar membrane contacts in parasite–host interaction.

#### 3.2.2 Role of Host Golgi Complex in Cholesterol Acquisition by the Amastigotes

Cholesterol homeostasis in the cells is strictly controlled by the ER, and the cholesterol gradient ranges from low concentration at the ER to higher concentration at the PM. Cholesterol at the ER is transported to the Golgi via non‐vesicular transport and is enriched at the trans‐Golgi (Ikonen and Olkkonen 2023), from where any vesicles bud off as part of the secretory pathway. Based on the role of the Golgi apparatus in the secretion of molecules, we also analyze its contribution to cholesterol traffic to amastigotes.

**FIGURE 7:**
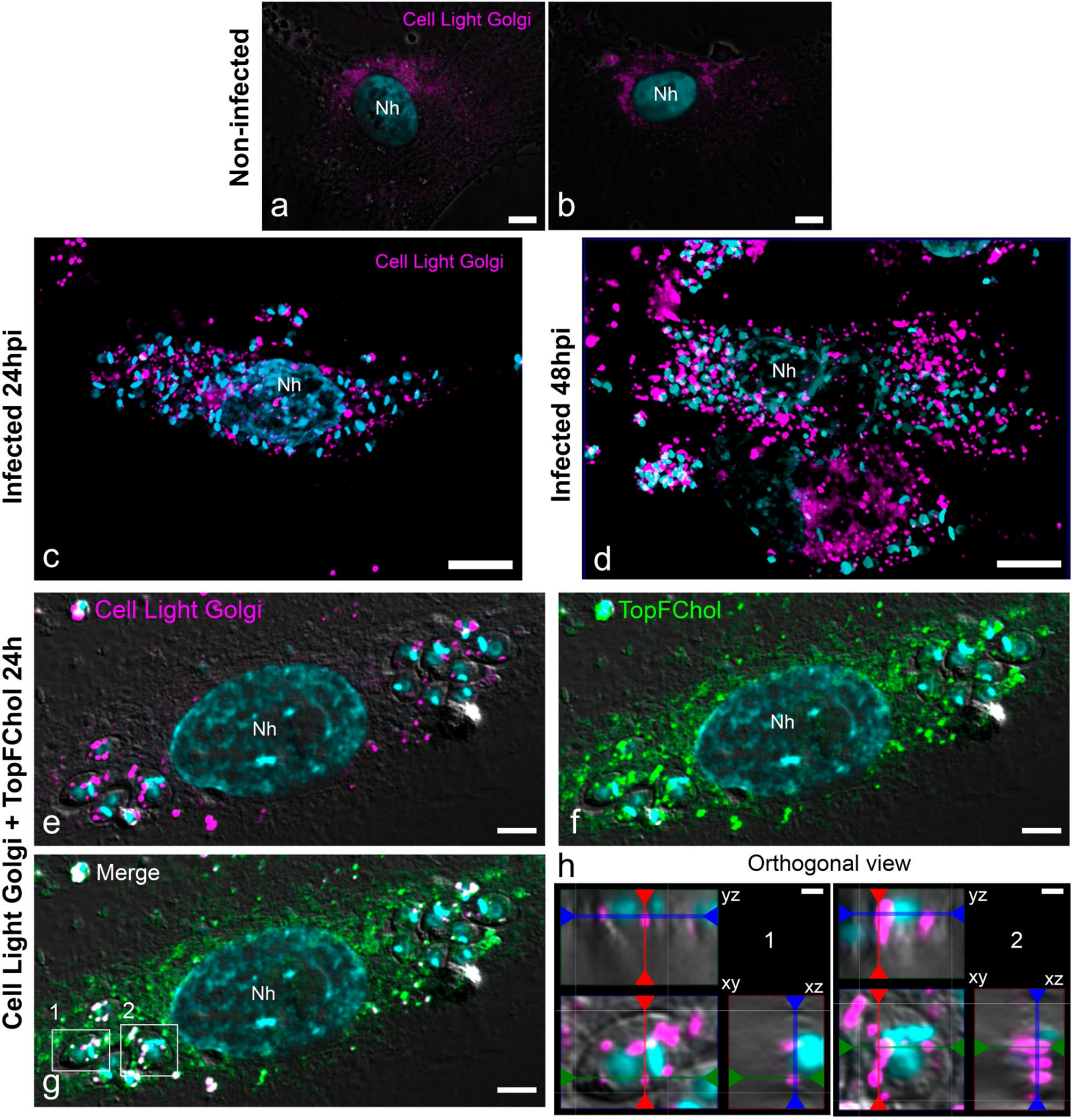
Host Golgi complex remodeling and participation in TopFChol delivery to amastigotes. Non‐infected or infected cells HFF1 cells were incubated with Cell Light Golgi for 24 h. (a, b) Maximum intensity projection from *z*‐stack confocal series of non‐infected cells showed continuous labeling (magenta) restricted to the perinuclear region. Infected cells incubated with Cell Light Golgi 24 hpi (c) and 48 hpi (d) showed dispersed punctual labeling throughout the cytoplasm, in between the amastigotes. (e–h) Infected cells were incubated with Cell Light Golgi and then with TopFChol for 24 h. (e–g) Single plane of a *z*‐stack confocal series showed punctual labeling of Cell Light Golgi (e) in locations close or inside the amastigotes, and TopFChol signal (f) with regions of superposition between the two (g). (h) Orthogonal views of amastigotes highlighted by the rectangles 1 and 2 in (g). The white lines delimitate the boundaries of amastigote membrane. Cell light Golgi labeling is contained inside amastigotes in punctual structures from anterior to anterior of the parasite. Bars: a–d—10 μm; e–g—5 μm; h—1 μm.

We first compared Golgi distribution in non‐infected and infected cells using the reagent Cell Light Golgi‐RFP. In this case, the construct encodes a human trans‐Golgi resident enzyme, N‐acetylgalactosaminyltransferase, tagged with RFP. In vertebrate cells, the Golgi apparatus is arranged in multiple stacks that associate laterally, forming the Golgi ribbon, located perinuclearly (Ladinsky et al. 1999; Saraste and Prydz 2019). We observed that, in non‐infected cells cultivated with 10% delipidated FBS, Cell Light Golgi signal was localized to the perinuclear region (Figure 7a,b). In infected cells, 24 hpi (Figure 7c) and 48 hpi (Figure 7d), Cell Light Golgi signal labeled punctate structures throughout the cytoplasm, suggesting a fragmentation of the Golgi ribbon and dispersion from the perinuclear region. Then, based on our previous results, incubation of cells with TopFChol directly into the culture medium was the faster way for cholesterol to reach the amastigotes. In this context, cholesterol should be sequestered from PM by the ER and then transferred to the Golgi complex. Therefore, we incubated infected cells with Cell Light Golgi and, after 24 h, added TopFChol for an additional 24 h (Figure 7e,h). We observed punctual labeling of Cell Light Golgi (Figure 7e) localized at the cell cytosol and inside amastigotes that colocalized with the TopFChol stain (Figure 7f,g). Orthogonal views of different amastigotes confirm the localization of Cell Light Golgi inside them. These observations suggest that the Golgi complex may also contribute to cholesterol traffic, as evidenced by colocalization of Golgi markers and cholesterol tracer near or within the parasite.

**FIGURE 8:**
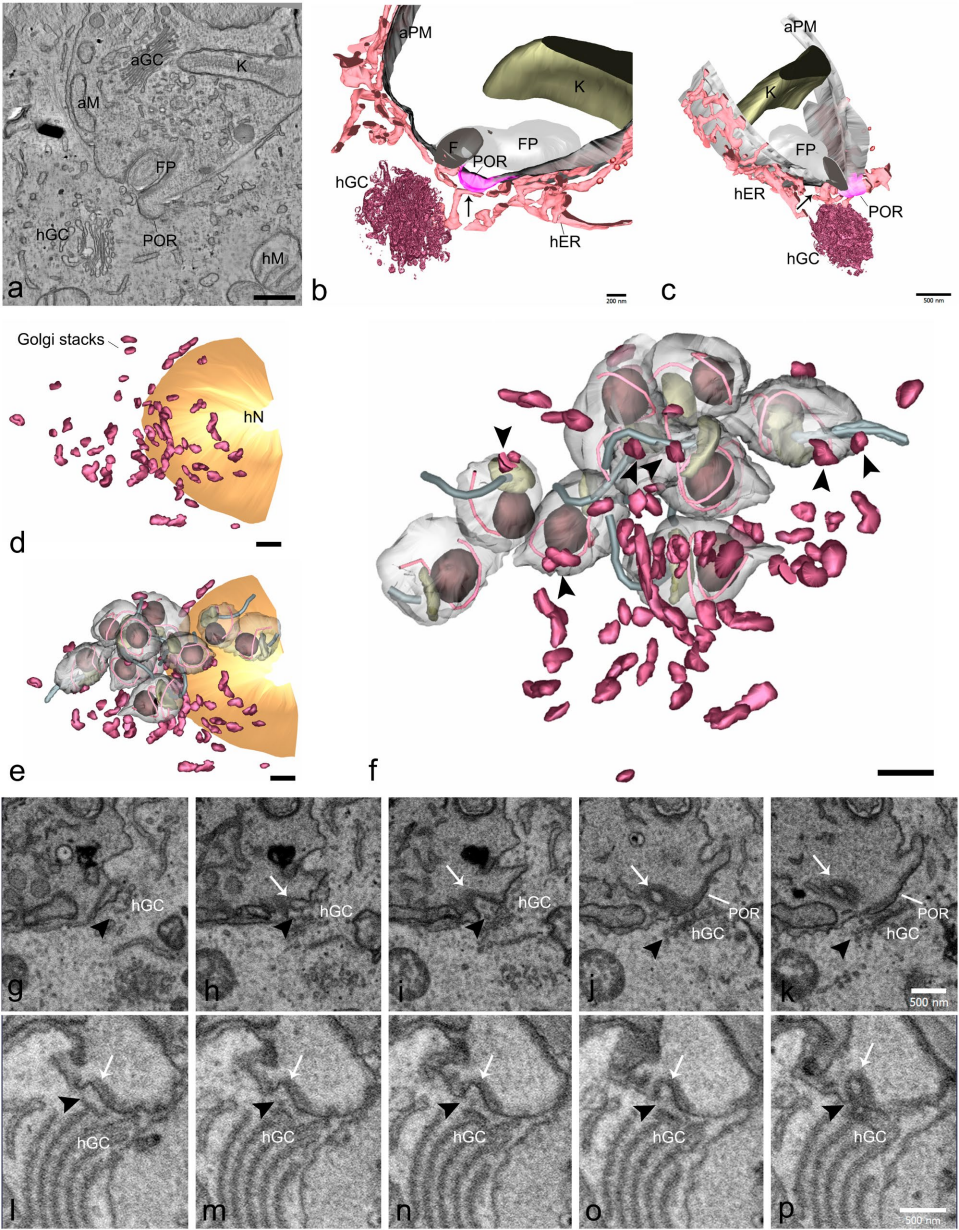
Host Golgi complex fragments during infection. HFF1 cells were infected with trypomastigotes and incubated with the growth medium supplemented with 10% of complete FBS (control) for 48 h. Cells were then fixed and processed for TEM. (a) Virtual slice of serial dual‐axis tomogram showing the proximity of a Golgi stack (hGC) to the anterior region of the amastigote, close to the preoral ridge (POR) and flagellar pocket (FP). (b, c) Different views of the 3D model produced from the serial tomogram are shown in (a). (b) Host ER (hER, pink) surrounds the amastigote membrane (aPM, gray) along the entire volume. Host Golgi was placed close to the amastigote POR and FP. An ER profile placed close to the Golgi could be seen touching the POR membrane domain (arrow). (c) ER, tethered the amastigote membrane along all reconstructed volumes. An ER profile could be seen close to the FP (arrow). (d–p) A slice‐and‐view series was obtained in a FIB‐SEM. (d–f) 3D reconstruction of the volume analyzed showing Golgi stacks (magenta) dispersed through the host cell cytoplasm (d). Golgi stacks were found scattered among the amastigotes (e, f). Golgi stacks get close to the amastigote membrane, especially at the anterior region of the parasite (arrowheads in f). (g–p) Sequential images of the FIB‐SEM series showing the region of contact of two different amastigotes (g–k, l–p) with a host Golgi stack (hGC, arrowheads). The cytostome is pointed by the arrow. Note the presence of Golgi‐derived vesicles at the entrance of the cytostome (arrowheads in h, i). Bars: a, c, g–p—500 nm; d–f—2 μm.

By using serial electron tomography and FIB‐SEM, we were able to get a tridimensional overview of the interaction between the host Golgi and amastigotes. We acquired three serial tomograms (four sections of 200 nm each—pixel size: 1.94 nm) of infected cells 48 hpi incubated in culture medium supplemented with 10% FBS. The tomogram shown in Figure 8a reveals the proximity of the host Golgi stack to the anterior region of amastigotes, close to the flagellar pocket and the preoral ridge (POR). The 3D reconstruction of the tomogram volume shows the distribution and proximity of the host ER to the amastigote membrane, which surrounds the amastigote entirely (Figure 8b,c, Movie S1). Despite the high resolution offered by electron tomography, the cellular volume that can be assessed by serial electron tomography is still limited. Therefore, we used FIB‐SEM to acquire an automated serial section from preparations of infected cells 48 hpi. The reconstructed volume was 6000 μm^3^. In Figure 8d,e, the tridimensional reconstruction shows an overview of the Golgi stacks distribution in infected cells, corroborating our findings in fluorescence microscopy concerning the fragmentation of the Golgi ribbon and dispersion of the Golgi stacks. The Golgi stacks were distributed among the amastigotes. Moreover, we observed that the proximity of Golgi elements with the amastigote membrane was found mainly at the anterior region of the parasite, close to the cytostome and the flagellar pocket (Figure 8f). Despite the low resolution of the FIB‐SEM technique, the volume imaged allowed us to analyze the ultrastructure of the interaction between the amastigotes and the host organelles, and snapshot images that suggest internalization of host vesicles derived from Golgi or ER through the cytostome‐cytopharynx complex (Figure 8g,p). This result shows that the structural organization of the Golgi apparatus in the host cells is modified after infection, with Golgi fragmentation in small stacks and dispersion through the cytosol, facilitating its contact with the amastigotes.

## 4 Discussion

Previous work had investigated the distribution of host cell organelles at the initial steps of *T. cruzi* infection and parasitophorous vacuole formation in non‐phagocytic (Tardieux et al. 1992) and phagocytic cells (Reignault et al. 2019) and evidenced the essential role of lysosomes, Golgi complex, and mitochondria. After escaping to the cytosol and differentiating, amastigotes were registered at the host perinuclear region in several host cell types (Lentini et al. 2018). In that situation of initial infection, amastigotes were found surrounded by endoplasmic reticulum and mitochondria. The authors chose to investigate the relationship with the mitochondria, finding contact sites with the parasite flagellar tip that they hypothesized would function as sensors (Lentini et al. 2018).

Understanding the mechanisms by which *T. cruzi* amastigotes scavenge nutrients from the host cell is important to unveil the factors that can lead to disease progression, particularly in the chronic phase, and for the development of strategies that can inhibit parasite replication.

Sterols are essential lipids in cellular membranes. They can have several important functions that include the maintenance of the liquid–solid phase of the membranes, allowing for signaling and membrane traffic inside the cells, as well as energy sources and reservoirs. Different types of sterols can be synthesized among living organisms. *Trypanosoma cruzi*, like other trypanosomatids and fungi, mainly produces ergostane‐type sterols (Urbina 2009), which differ from its vertebrate host, in which cholesterol is the major sterol. Because of that, the biosynthetic pathway for the production of ergosterol has been explored as a chemotherapy target for drug development (Kessler et al. 2013).

Although *T. cruzi* synthesizes its own sterols, it has been shown that epimastigotes are able to capture cholesterol from the extracellular medium through endocytosis of LDL (Soares and de Souza 1991). The endocytosed cholesterol can be distributed throughout the cell membranes of the parasite and also stored as cholesterol esters in lipid droplets or lipid inclusions inside reservosomes (Pereira et al. 2011, 2018, 2015). The main carbon source used by *T. cruzi* for energy production is still a matter of debate. However, some data from the literature point to the important role of lipids as energy fuel (Gazos‐Lopes et al. 2017).

Amastigotes grow and replicate inside the cytosol of host vertebrate cells. Differently from other intracellular protists, such as *Leishmania* sp. and *Toxoplasma gondii*, that proliferate inside a parasitophorous vacuole (Batista et al. 2020), amastigotes are free in the cytosol, having direct access to the host cytosol macromolecules and organelles. In this context, it has been shown that amastigotes possess more than 80% by weight of their sterol as cholesterol (Liendo et al. 1999), suggesting that they may scavenge this lipid from the host cell.

In this work, we demonstrated, for the first time, the ability of amastigotes to capture cholesterol from the host cell. We used a fluorescent cholesterol analog, TopFluor Cholesterol (or BODIPY‐cholesterol), whose biophysical properties were shown to be very similar to cholesterol, especially for lipid traffic assays (Holtta‐Vuori et al. 2016). For that, we tested different methods of delivering TopFChol to cells to study the impact of the traffic dynamics on the parasite.

The observation that cholesterol was internalized by amastigotes faster when TopFChol was added directly to the growth medium when compared with TopFChol provided inside LDL particles suggested that amastigotes intercept cholesterol along the pathway from the host cell plasma membrane to intracellular membranes prior to its passage through the host's endocytic route. The different dynamics for cholesterol internalization were described in models of lysosomal cholesterol accumulation using NPC1‐deficient fibroblasts (Holtta‐Vuori et al. 2016). They showed that, when incubated in the medium, TopFChol took more than 18 h to start to accumulate in lysosomes, while when provided inside LDL particles, it took just 2 h. This suggests that in the case of amastigotes, access to TopFChol may happen downstream of lysosomal export. In this context, the role of the host ER emerges as an important factor in this traffic. This is not a surprise since the ER is the site of cholesterol synthesis and a major regulator of cholesterol homeostasis in the cell. Previous work estimated that approximately 30% of the LDL‐derived cholesterol is exported to the ER without traffic through the plasma membrane (Neufeld et al. 1996).

Cholesterol signals in amastigotes were characterized by punctual labeling along the anterior/posterior region of the parasite, with major concentration at the anterior region, close to the flagellar pocket. In most trypanosomatids of medical importance, the flagellar pocket is the sole site of all endo‐exocytosis (Halliday et al. 2020). However, *T. cruzi* possesses an additional structure related to endocytosis, the cytostome‐cytopharynx complex (Alcantara et al. 2014). This long membrane invagination opens at the cell surface, close to the flagellar pocket, and is separated from it by a highly glycosylated membrane domain called Preoral Ridge. We have shown that the preoral ridge domain extends from inside the flagellar pocket toward the cytostome and that it may participate in the transport of endocytic cargo that binds to the flagellar pocket membrane to the cytostome where it can be endocytosed (Alcantara et al. 2014). The concentration of cholesterol signals at the anterior region of the amastigotes could indicate the participation of the endocytic portals in its internalization. Indeed, immunogold labeling of infected cells incubated with TopFChol showed strong labeling for the cholesterol at the preoral ridge region. Moreover, the use of MyoF‐tagged parasites, a protein localized in the cytostome‐cytopharynx complex and that participates in the endocytic traffic of the parasite, shows clearly that cholesterol capture occurs through the cytostome‐cytopharynx complex.

Cholesterol intracellular transport in mammalian and yeast cells, where it is best characterized, possesses two main mechanisms: vesicular traffic and non‐vesicular traffic. Non‐vesicular lipid transport is mediated by membrane contact sites (MCS), regions of close membrane apposition (5–30 nm) between neighboring organelles (Reinisch and Prinz 2021), and accounts for a fast route of lipid exchange. The lipid traffic between membranes is mediated by lipid transfer proteins (LTPs) that can act either as shuttles or bridges, helping to tether membranes and facilitate lipid transfer (Wong et al. 2019). EM has been considered the “gold standard” method for the visualization and characterization of MCSs (Huang et al. 2020). For that, we resorted to the use of electron tomography (ET) and FIB‐SEM to characterize the mechanism of cholesterol uptake by amastigotes. Our results indicate the participation of MCS between ER and amastigotes as a probable mechanism of cholesterol traffic between the host cell and the parasite. Multiple bridges between the ER and the amastigote membrane were seen by ET whose dimensions are compatible with what was previously described for these bridges in intraorganellar MCS.

Besides ER, the host Golgi complex seems also to contribute to the traffic of cholesterol to amastigotes. We observed Golgi ribbon fragmentation and stack dispersion upon infection with *T. cruzi*, highlighting a remodeling of the organelle never explored before in *T. cruzi* ‐host interaction. Golgi fragmentation is also observed in other pathogenic diseases; the induction of Golgi fragmentation seems to be actively mediated by the pathogens (Liu et al. 2021). In *Toxoplasma gondii* infection, Golgi fragmentation and dispersal around the parasitophorous vacuole are important for the acquisition of host sphingolipids by the parasite, by sequestering host Golgi‐derived vesicles (Romano et al. 2013). By analyzing a big volume from an infected cell, we could see the approximation of the host Golgi stacks with the anterior region of amastigotes. The observation of Golgi‐derived vesicles close to the entrance of the cytostome suggests that endocytosis of Golgi vesicles could occur. This could explain some previous data from the literature that showed uptake of host TGF‐β by amastigotes infecting cardiomyocytes (Waghabi et al. 2005). In this work, Waghabi and colleagues observed, by immunostaining host TGF‐β, labeling inside amastigotes but not in trypomastigotes. TGF‐β is a cytokine that is secreted by the cells. This way, its production follows the classical biosynthetic‐secretory pathway of the cell, being translated in ER, transferred to Golgi, and secreted to the plasma membrane via Golgi‐secreted vesicles. In that work, the major question that remained was how amastigotes had access to this cytokine if it is contained inside secretory vesicles, and one of the hypotheses was the ability of amastigotes to endocytose host secretory vesicles. Corroborating this, we also showed the presence of ER‐ proteins and Golgi proteins inside amastigotes, not only by antibody staining of these proteins but also by using expression vectors to produce tagged proteins. Our data consistently showed that these proteins can be internalized by the amastigotes, probably via the endocytic route.

Although this work does not identify the proteins responsible for the cholesterol traffic into ER‐amastigotes MCS, we brought important mechanistic insights into the pathways that *T. cruzi* amastigotes can use to scavenge host molecules. From here, we can now have a clue about the proteins that may participate in these MCS, especially proteins derived from the parasite. It is important to point out that *T. cruzi* may have lipid transfer proteins localized at its plasma membrane that can deal with cholesterol scavenging from host ER and Golgi. Of note, from what we know about *T. cruzi* cellular physiology, the cholesterol traffic from the parasite cell surface to intracellular membranes must be dependent on the parasite endocytic pathway, since subpellicular microtubules preclude the contact of any intracellular organelle with the parasite plasma membrane (Zuma et al. 2021). This way, cholesterol inserted into the membrane should be delivered to the cytostome‐cytopharynx complex by lateral diffusion and internalized by endocytosis, after vesicle budding from the cytopharynx.

The relevance of cholesterol scavenging from the host became clear with our demonstration that host cholesterol availability directly impaired amastigote growth and development. The presence of cholesterol in the medium was essential to support amastigote growth. Incubation with delipidated FBS decreased amastigote development, suggesting that *de novo* synthesis of cholesterol by the host cell was not sufficient to fulfill the sterol requirements for amastigotes. Addition of TopFChol directly to the culture medium was able to restore amastigote growth to levels like the control (cells supplemented with 10% FBS). The total cholesterol concentration in FBS ranges from 300 to 500 μg/mL (Else 2020). That means the concentration of total cholesterol in the culture medium supplemented with 10% FBS was around 30–50 μg/mL. In our assay, we used 100 μg/mL of TopFChol to supplement the delipidated FBS, which could explain not only the restoration of the amastigote growth but also the slightly higher growth when compared with the control. We acknowledge the possibility that the observed impact of cholesterol supplementation on *T. cruzi* amastigote growth and infection rate could, at least in part, be an indirect effect. The depletion of cholesterol from the host cell may impair cellular fitness, thereby reducing the quality of the intracellular niche available to the parasite. This could affect various host cell processes, such as membrane integrity, vesicular trafficking, and overall metabolic activity, which are crucial for amastigote survival and replication. While our data demonstrate a dependence of *T. cruzi* amastigotes on cholesterol, further experiments are needed to disentangle the direct effects on the parasite from the potential influence of host cell fitness. These experiments will aim to evaluate host cell viability and function under cholesterol‐depleted conditions, as well as explore whether cholesterol supplementation rescues any host cell deficits that may indirectly support parasite growth.

The role of cholesterol scavenging by amastigotes remains a topic of debate in the literature. Chemotherapy studies examining the effects of inhibitors on the sterol biosynthesis pathway may provide some insights into this question. Current findings indicate that inhibitors of lanosterol‐14α‐demethylase (CYP51) do not achieve a sterile cure in clinical settings (Molina et al. 2014; Torrico et al. 2018) or animal models (Khare et al. 2015), likely due to a decreased presence of amastigotes in tissues, which reduces the need for de novo sterol synthesis. In this context, cholesterol may act as a substitute for endogenous sterols. In epimastigotes, knocking out CYP51 and squalene epoxidase (SQLE) results in the complete loss of all endogenous sterols, with the only sterol present being exogenous cholesterol (Dumoulin et al. 2022). Although these mutants' growth is slower than that of wild‐type parasites, they remain viable. This suggests that exogenous cholesterol can adequately meet “the parasite's metabolic and structural sterol requirements”

The findings presented here complement previous reports describing the involvement of host lipoproteins in the pathogenesis of *T. cruzi* infection. It was demonstrated that *T. cruzi* has a high affinity for low‐density lipoprotein (LDL) and uses the LDL receptor (LDLr) for host cell invasion (Nagajyothi et al. 2011). Moreover, they showed that *T. cruzi* infection results in increased intracellular LDL and cholesterol, which accumulate in cardiac and adipose tissues during both acute and chronic phases (Johndrow et al. 2014). Our observations of LDL‐derived cholesterol incorporation into host intracellular compartments and parasite membrane domains are in line with these findings and further suggest that the endocytic and organelle‐associated pathways exploited by *T. cruzi* in vitro may also operate in vivo. Importantly, adipose tissue has been identified as a long‐term reservoir for *T. cruzi* , with infected adipocytes displaying altered lipid metabolism and increased inflammatory mediator expression (Nagajyothi et al. 2009).

Building on this body of work, our study provides the first solid evidence that intracellular amastigotes actively acquire host‐derived lipids, particularly cholesterol, and that this process directly impacts parasite fitness during the intracellular cycle. By showing that the supplementation or depletion of host cholesterol affects amastigote proliferation and survival, and by identifying structural and spatial associations between amastigotes and host organelles involved in lipid metabolism, our results advance the understanding of how *T. cruzi* depends on host lipid resources to sustain infection. These findings also raise the possibility that therapeutic targeting of lipid acquisition pathways could impair intracellular parasite development.

Figure S4 summarizes our results and hypotheses. Based on our imaging data, we propose that extracellular‐derived cholesterol may reach amastigotes through multiple host cell pathways. The observed proximity and membrane contact between host organelles (such as ER and Golgi) and the amastigote plasma membrane support a model in which cholesterol trafficking could involve both vesicular and non‐vesicular interactions, although functional validation is still needed.

## Supporting information


**Movie S1.** Virtual sections of a dual‐axis serial tomogram of HFF1 cells 48 hpi. Cells were grown in a culture medium supplemented with 10% of complete FBS (control). The tomogram shows an area of close contact between the host ER and Golgi stack with the anterior region of the amastigote membrane.

**Figure S1.** Dynamics of TopFChol internalization by intracellular amastigotes through incubation with LDL‐TopFChol and Cell Light ER‐RFP. HFF1 cells were infected with trypomastigotes and, after 24 h, incubated with Cell Light ER‐RFP for 24 h. LDL‐TopFChol was then added to a growth medium supplemented with 10% dFBS and incubated for 4, 24, and 48 h. Single‐plane fluorescence images from the *z*‐stack confocal series are shown. Colocalization of the TopFchol signal and Cell Light ER‐RFP was observed inside amastigotes at all time points. At 48 h, many colocalizing punctual labeling was observed. Bars: 5 μm.

**Figure S2.** Dynamics of TopFChol internalization by intracellular amastigotes through incubation with TopFChol directly to the culture medium and Cell Light ER‐RFP. HFF1 cells were infected with trypomastigotes and, after 24 h, incubated with Cell Light ER‐RFP for 24 h. TopFChol was then added to a growth medium supplemented with 10% of dFBS and incubated for 4, 24, and 48 h. Single‐plane fluorescence images from the *z*‐stack confocal series are shown. TopFChol labeling inside amastigotes has been registered since 4 h of incubation. TopFChol signal colocalized with Cell Light ER‐RFP inside amastigotes at all time points. At 48 h, many colocalizing punctual labeling was observed. Bars: 5 μm.

**Figure S3.** Differences in host cell organelle distribution around amastigotes in different conditions of lipids availability. HFF1 cells were infected with trypomastigotes and, 24 h after, the growth medium was changed, and cells were incubated with 10% of complete FBS (control) or with 10% dFBS (lipid deprivation). Cells were fixed 48 h later and processed for TEM. (a–d) TEM images of cells incubated with complete FBS. (a) Host lysosomes (L, green) and ER (pink) could be seen very close to the amastigote membrane. (b) Higher magnification of the region highlighted by the rectangle in (a). Lysosomes showed direct contact with the amastigote membrane (arrowhead). (c) ER appeared to embrace amastigotes at some points of contact (arrows). (d) Higher magnification of the region highlighted by the rectangle in (c). ER profiles could be seen touching the amastigote membrane at the region of the preoral ridge (POR) and the cytostome (Cy) (arrows). (e–i) TEM images of cells incubated with 10% dFBS. (e) Low magnification TEM image showing an infected cell full of amastigotes (blue), prominent ER, and a few lysosomes (green). (f–i) ER profiles could be seen touching the membrane of amastigotes (arrows) at many points, especially at the amastigote anterior region (g–i). aM, amastigote mitochondria; aN, amastigote nucleus; F, flagellum; hM, host mitochondria; hN, host nucleus; K, kinetoplast. Bars: a, c, f—2 μm; b, d, g—500 nm; e—5 μm; h, i—1 μm.

**Figure S4.** Schematic representation of the routes of cholesterol transport to amastigotes. (1) cholesterol loaded in LDL particles is captured by receptor‐mediated endocytosis (1a), passing through the early endosomes and late endosomes (1b) and being released in the lysosomes (1c), from where it can be transferred to ER and then to amastigotes via MCSs (1d) or it can be transferred directly from lysosomes to amastigotes via MCSs (1e). (2) Cholesterol in excess in the PM is equilibrate by ER‐PM MCS establishment (2a), which then can be accessed by amastigotes via ER‐amastigotes MCSs (2b). Cholesterol in excess in the ER can be transferred to fragmented Golgi stacks via vesicular transport or from MCSs, and Golgi‐amastigotes MCSs may be responsible for cholesterol transfer to amastigotes (2c). Another alternative is the direct endocytosis of Golgi‐derived vesicles by amastigotes (2d). Amastigote cytostome‐cytopharynx complex (Cyt) may concentrate shuttled cholesterol and internalize it through endocytosis. aN, amastigote nucleus; hN, host nucleus; K, kinetoplast; POR, preoral ridge.

## Data Availability

Research data are not shared.
