# Supplementary figures and images for: Host Organelle Interactions Facilitate Cholesterol Acquisition by Trypanosoma cruzi Amastigotes
Source: J Eukaryot Microbiol. 2025 Jul 20;72(4):e70027. doi: 10.1111/jeu.70027 (PMC12277875; doi:10.1111/jeu.70027)

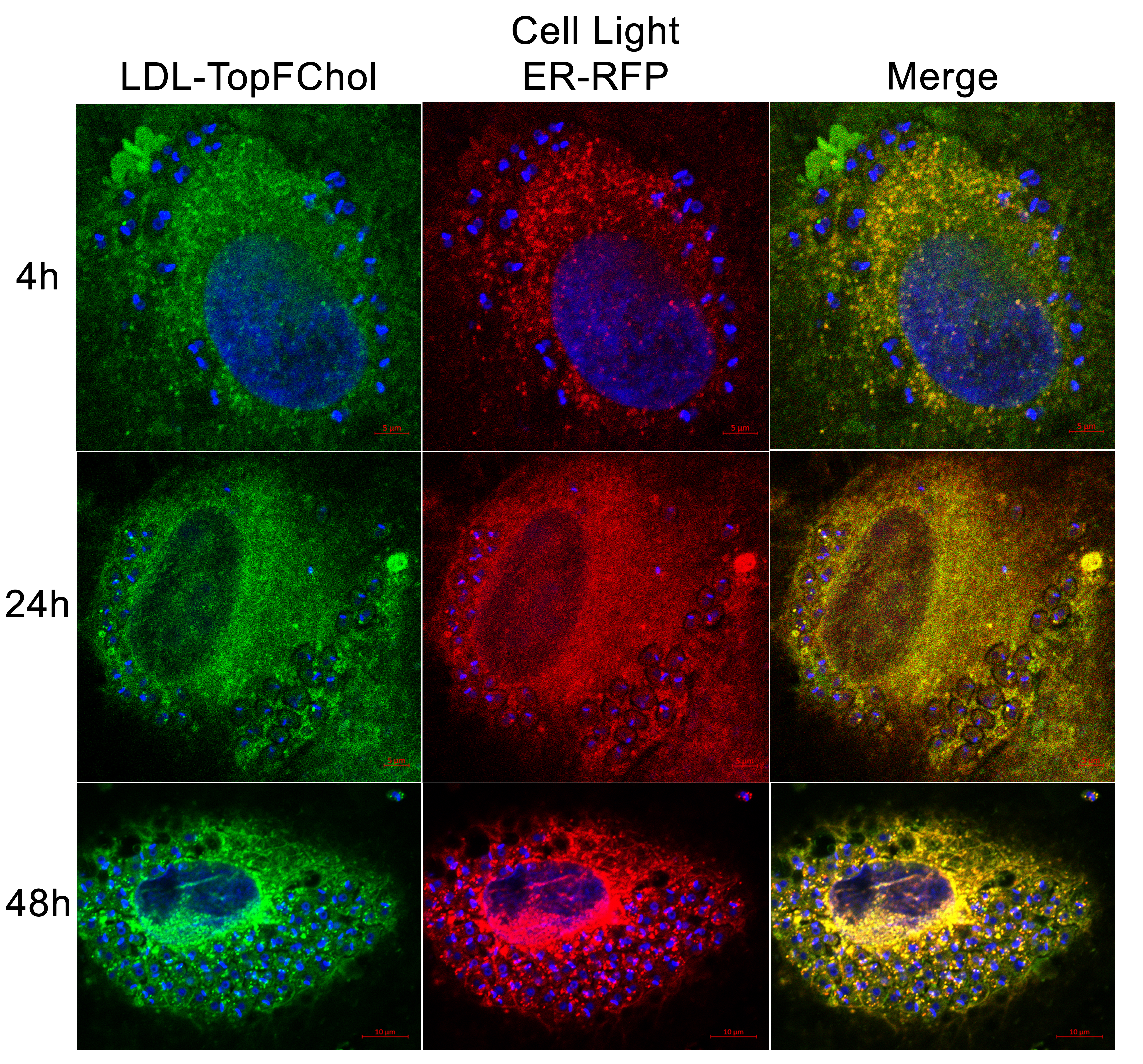

Supplement: Supplementary file 2 — Figure S1. Dynamics of TopFChol internalization by intracellular amastigotes through incubation with LDL‐TopFChol and Cell Light ER‐RFP. HFF1 cells were infected with trypomastigotes and, after 24 h, incubated with Cell Light ER‐RFP for 24 h. LDL‐TopFChol was then added to a growth medium supplemented with 10% dFBS and incubated for 4, 24, and 48 h. Single‐plane fluorescence images from the z‐stack confocal series are shown. Colocalization of the TopFchol signal and Cell Light ER‐RFP was observed inside amastigotes at all time points. At 48 h, many colocalizing punctual labeling was observed. Bars: 5 μm. [file JEU-72-e70027-s004.jpg]

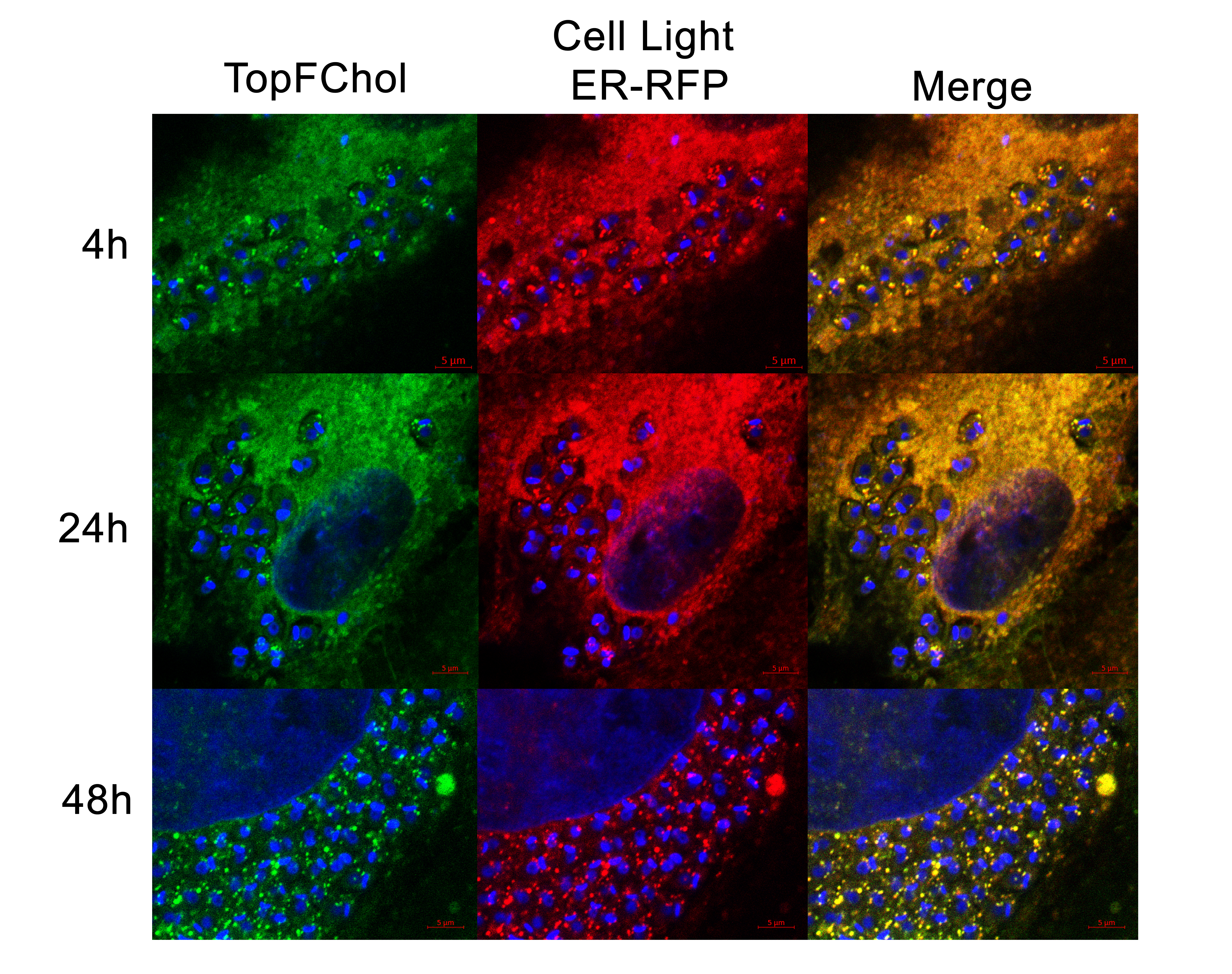

Supplement: Supplementary file 3 — Figure S2. Dynamics of TopFChol internalization by intracellular amastigotes through incubation with TopFChol directly to the culture medium and Cell Light ER‐RFP. HFF1 cells were infected with trypomastigotes and, after 24 h, incubated with Cell Light ER‐RFP for 24 h. TopFChol was then added to a growth medium supplemented with 10% of dFBS and incubated for 4, 24, and 48 h. Single‐plane fluorescence images from the z‐stack confocal series are shown. TopFChol labeling inside amastigotes has been registered since 4 h of incubation. TopFChol signal colocalized with Cell Light ER‐RFP inside amastigotes at all time points. At 48 h, many colocalizing punctual labeling was observed. Bars: 5 μm. [file JEU-72-e70027-s002.jpg]

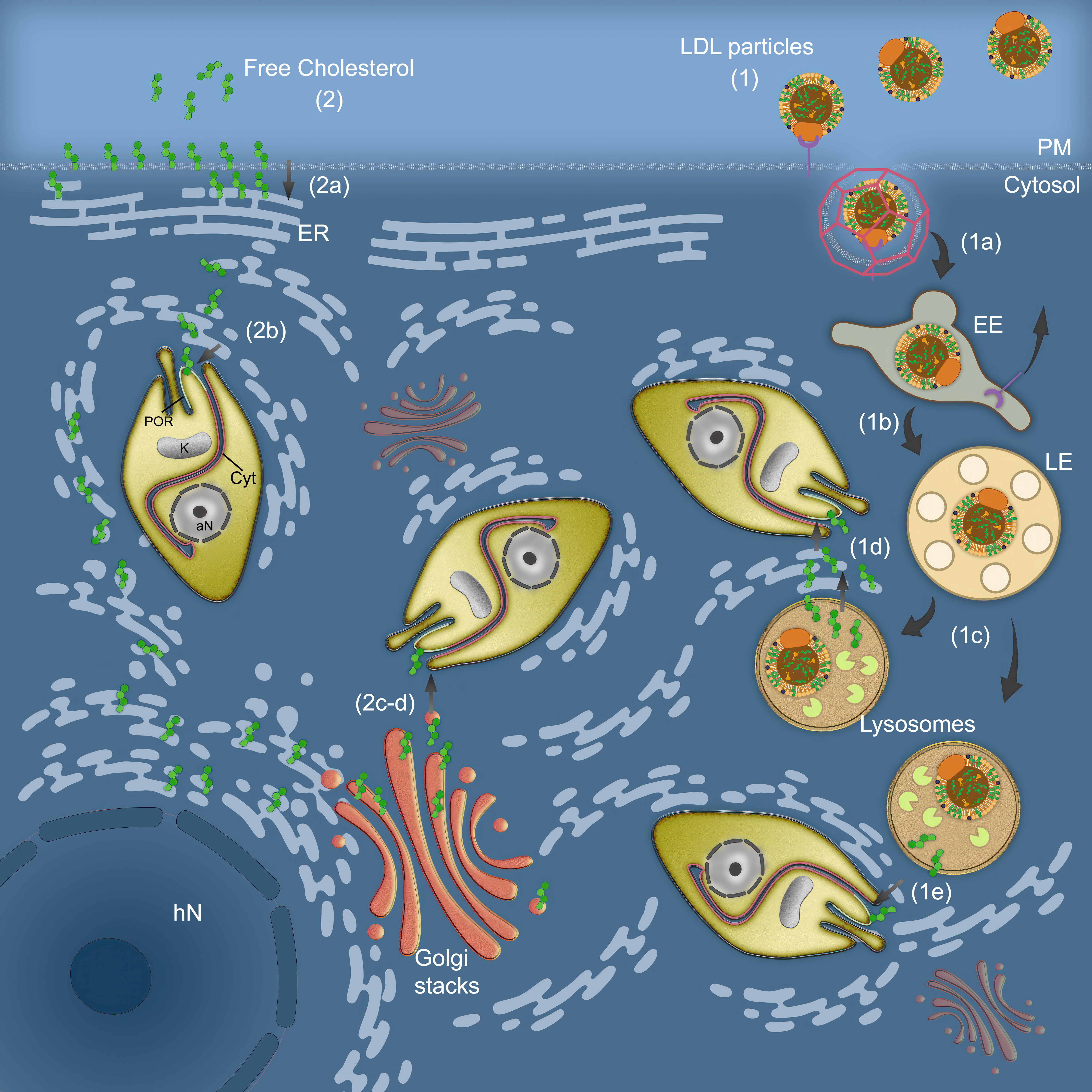

Supplement: Supplementary file 5 — Figure S4. Schematic representation of the routes of cholesterol transport to amastigotes. (1) cholesterol loaded in LDL particles is captured by receptor‐mediated endocytosis (1a), passing through the early endosomes and late endosomes (1b) and being released in the lysosomes (1c), from where it can be transferred to ER and then to amastigotes via MCSs (1d) or it can be transferred directly from lysosomes to amastigotes via MCSs (1e). (2) Cholesterol in excess in the PM is equilibrate by ER‐PM MCS establishment (2a), which then can be accessed by amastigotes via ER‐amastigotes MCSs (2b). Cholesterol in excess in the ER can be transferred to fragmented Golgi stacks via vesicular transport or from MCSs, and Golgi‐amastigotes MCSs may be responsible for cholesterol transfer to amastigotes (2c). Another alternative is the direct endocytosis of Golgi‐derived vesicles by amastigotes (2d). Amastigote cytostome‐cytopharynx complex (Cyt) may concentrate shuttled cholesterol and internalize it through endocytosis. aN, amastigote nucleus; hN, host nucleus; K, kinetoplast; POR, preoral ridge. [file JEU-72-e70027-s005.jpg]
